# People With Prior Tuberculosis Have Accelerated Frailty and Differential Survival into Older Age: A Cross-sectional Analysis in Southern Africa

**DOI:** 10.1093/infdis/jiag299

**Published:** 2026-06-12

**Authors:** M Coleman, A Manyara, C J Calderwood, A de Wilton, E I Y Madela, H Wilson, A Burton, L Gates, B Cassim, C Pearse, T Madanhire, R A Ferrand, K A Ward, K Kranzer, C L Gregson

**Affiliations:** Sydney Infectious Diseases Institute, University of Sydney, Sydney, New South Wales, Australia; Institute of Infectious Diseases and Tropical Medicine, University Hospital, LMU Munich, Munich, Germany; Global Health and Ageing Research Unit, Bristol Medical School, University of Bristol, Bristol, United Kingdom; Clinical Research Department, London School of Hygiene and Tropical Medicine, London, United Kingdom; The Health Research Unit Zimbabwe, Biomedical Research and Training Institute, Harare, Zimbabwe; National Heart and Lung Institute, Imperial College London, London, United Kingdom; Global Health and Ageing Research Unit, Bristol Medical School, University of Bristol, Bristol, United Kingdom; Department of Geriatrics, University of KwaZulu-Natal, Durban, KwaZulu-Natal, South Africa; Global Health and Ageing Research Unit, Bristol Medical School, University of Bristol, Bristol, United Kingdom; Global Health and Ageing Research Unit, Bristol Medical School, University of Bristol, Bristol, United Kingdom; MRC Lifecourse Epidemiology Centre, Human Development and Health, University of Southampton, Southampton, United Kingdom; Department of Geriatrics, University of KwaZulu-Natal, Durban, KwaZulu-Natal, South Africa; MRC Lifecourse Epidemiology Centre, Human Development and Health, University of Southampton, Southampton, United Kingdom; Global Health and Ageing Research Unit, Bristol Medical School, University of Bristol, Bristol, United Kingdom; Clinical Research Department, London School of Hygiene and Tropical Medicine, London, United Kingdom; The Health Research Unit Zimbabwe, Biomedical Research and Training Institute, Harare, Zimbabwe; MRC Lifecourse Epidemiology Centre, Human Development and Health, University of Southampton, Southampton, United Kingdom; MRC Unit the Gambia, London School of Hygiene and Tropical Medicine, Banjul, The Gambia; Institute of Infectious Diseases and Tropical Medicine, University Hospital, LMU Munich, Munich, Germany; Clinical Research Department, London School of Hygiene and Tropical Medicine, London, United Kingdom; The Health Research Unit Zimbabwe, Biomedical Research and Training Institute, Harare, Zimbabwe; Global Health and Ageing Research Unit, Bristol Medical School, University of Bristol, Bristol, United Kingdom; The Health Research Unit Zimbabwe, Biomedical Research and Training Institute, Harare, Zimbabwe

**Keywords:** tuberculosis, healthy ageing, frailty, post-TB, multimorbidity

## Abstract

Post-tuberculosis disability impairs quality-of-life, yet chronic functional outcomes for tuberculosis survivors remain under-characterized. This cross-sectional sub-study (2022–2024) estimated Fried frailty phenotype prevalence among adults aged ≥40 years with self-reported prior tuberculosis in South Africa and Zimbabwe, compared with those with no tuberculosis history. Survivor bias was evaluated using historic WHO data. After adjusting for age, sex, education, HIV, diabetes and socioeconomic factors, prior tuberculosis was associated with twice the odds of frailty (OR = 2.06, 95% CI 1.38–3.03, *P* = .001), and worse survival into older age. As longevity increases in Southern Africa, healthy-ageing interventions for tuberculosis survivors could improve function and survival into older age.

## Teaser

Longer-term effects of tuberculosis into older age are poorly understood. In Southern Africa, older adults self-reporting prior tuberculosis had twice the odds of frailty compared with people without tuberculosis history, after adjusting for age, sex, HIV, diabetes, and socioeconomic factors and were less likely to survive to older age.

Tuberculosis continues to cause more deaths globally than any other infectious disease [1]. Among those who survive pulmonary tuberculosis, destruction of lung architecture and chronic inflammation results in long-term respiratory disability [2]; however, post-tuberculosis impairments likely extend beyond the lungs [2] and may accelerate frailty.

Frailty is a state of reduced physiological resilience, which increases vulnerability to even minor stressors. With frailty, functional ability declines and mortality increases [3]. Chronic respiratory disease is an established risk factor for frailty [4]. People with chronic obstructive pulmonary disease (COPD) had 50% higher odds of frailty and prefrailty compared with those without COPD in one longitudinal study [5]. Pulmonary tuberculosis is a strong risk factor for COPD and bronchiectasis [2]. Parallels between the pathophysiology and manifestations of chronic respiratory diseases—including COPD, bronchiectasis, fibrosis, and post-tuberculosis lung disease [6]—could suggest similar mechanisms for post-tuberculosis-mediated functional decline, such as chronic systemic inflammation and sarcopenia [4]. Despite these parallels, the relationship between post-tuberculosis and frailty in adults remains unexplored.

While most common in adults over 65 years, frailty can occur in younger people and it can be mitigated with intervention, especially at early prefrail stages [7]. In the African region, prevalence of frailty substantially exceeds global norms [8]. While evidence is mixed and reasons remain unclear, chronic inflammation from coinfections such as Human Immunodeficiency Virus (HIV) may contribute [9]. Examining whether prior tuberculosis, common in the African region [1], predicts or exacerbates frailty may help identify a risk group who would benefit from intervention. In Southern Africa at the turn of the 21st century and prior to antiretroviral therapy being available in Africa, HIV mortality accounted for >40% of all-cause deaths; those who survived are now older, many having had tuberculosis [10]. Understanding the effect of prior tuberculosis on frailty must also account for differential survival among those with and without HIV over these years.

We estimated the prevalence of frailty among adults aged ≥40 years who self-reported prior tuberculosis in South Africa and Zimbabwe, independent of socioeconomic, comorbid and lifestyle factors, and examined differential survival among people with prior tuberculosis.

## METHODS

### Study Design

This population-based cross-sectional sub-study included adults aged ≥40 years, residing in rural and peri-urban sites in Zimbabwe and South Africa who had been recruited into a wider musculoskeletal study between January 2022 and April 2024 [11]. Self-report of having ever been diagnosed with tuberculosis was ascertained by researcher-administered questionnaire, together with sociodemographic characteristics, comorbidities, and lifestyle factors. Frailty was determined using the contextualized five-point Fried frailty phenotype (exhaustion, weight loss, low physical activity, grip strength, and gait speed) [11], with the presence of 3 or more criteria defining frailty, 1 or 2 criteria defining prefrailty, and zero equating to robust (frailty definition in Supplementary Table 1) [3, 9].

### Statistical Analysis

Frailty, HIV, and prior tuberculosis (ie, ever having been diagnosed) prevalence by age decile were plotted using R 4.4.3. Univariable associations between those with and without prior tuberculosis were assessed by sex using Fisher's exact and Chi [2] tests. Multivariable logistic regression examined the relationship between prior tuberculosis and frailty, adjusting for age, sex, country, educational attainment, food insecurity, household wealth, HIV, diabetes, smoking, and alcohol consumption as common shared risk factors for tuberculosis and frailty [8]. Stratified analyses assessed effect modification by sex, age group, HIV status and known lung damage. We did not adjust for BMI as weight loss is a component of the frailty outcome variable.

Expected prior tuberculosis prevalence in South Africa and Zimbabwe was estimated by 10-year age band standardized to the study age distribution. Cumulative lifetime risk was calculated from WHO case notifications (1980–2023) [12] and population data [13] assuming all cases occurred in the population aged ≥15 years. Incidence before 1980 was estimated using the 1980–1985 mean. Estimates did not account for recurrence. Expected prevalence by age decile was compared with observed self-reported prior tuberculosis. Further detail in Supplementary Methods.

## RESULTS

Among 2995 adults, the median age was 60 years (interquartile range 50–71), 1590 were female (53.1%), 1862 (62.2%) had attained at least a secondary education, 1555 (51.9%) were food insecure, 1151 (38.4%) had low household wealth, 147 (5.0%) were underweight (BMI<18.5 kg/m^2^), 717 (24.0%) reported currently or formerly smoking, 820 (27.5%) consumed alcohol, 928 (31.0%) had tested positive for HIV (of whom 878 (94.6%) were established on ART), and 434 (14.5%) had diabetes based upon random glucose tests and existing medication (Supplementary Table 2). Of those included in the study, 271 (9.1%) had prior tuberculosis. Prevalence of prior tuberculosis was 6.4% among women and 12.0% among men and higher in South Africa than Zimbabwe (11.3% vs 5.2%). Overall, 409 (13.7%) adults were frail and 1766 (59.0%) prefrail.

Prior TB was associated with older age, living in South Africa, lower educational attainment, food insecurity, being underweight, living with HIV, current or former smoking and alcohol consumption, irrespective of sex (Supplementary Table 2**)**.

The prevalence of HIV (without prior tuberculosis), prior tuberculosis (without HIV), and both HIV and tuberculosis, declined with age, while frailty increased (Figure 1*A*) across age bands. Age bands included 689 (23.0%) people aged 40–49 years, 757 (25.3%) 50–59 years, 635 (21.2%) 60–69 years, 664 (22.2%) 70–79 years and 250 (8.3%) 80+ years. With age, the observed prevalence of prior tuberculosis diverged from the expected prevalence in both countries (Figure 1*B*).

**Figure 1.**
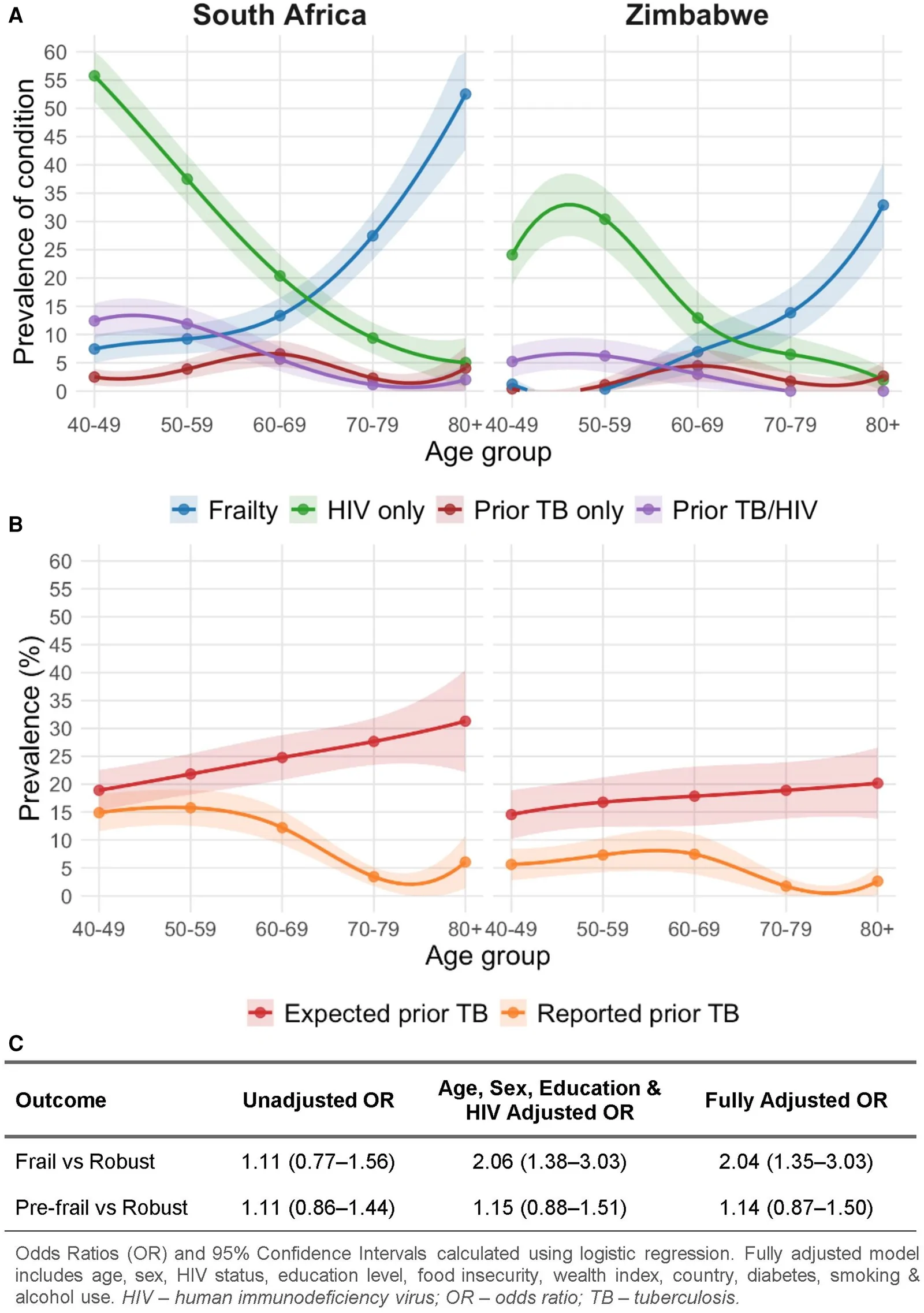
Reported and expected prior tuberculosis, HIV positive and frailty prevalence by age decile with adjusted multivariate logistic regression examining frail outcomes compared with robust among older adults self-reporting prior tuberculosis in South Africa and Zimbabwe. *A*, Prevalence of self-reported prior tuberculosis, composite Fried frailty status (“frailty”) and HIV test positive/prior tuberculosis self-report negative (“HIV only”) is plotted by age decile in South Africa and Zimbabwe, with self-report of prior tuberculosis disaggregated by HIV-associated (“prior TB/HIV”) and HIV-unassociated (“prior TB only”) prior disease. Standard error is indicated by faint color bands. *B*, Expected prevalence of prior tuberculosis (“expected prior TB”) is estimated as cumulative lifetime prevalence from ≥15 y based upon historical annual case notification rates and ≥15 population records by country, standardized to the age composition of study participants in each age decile. Prevalence of all self-reported prior tuberculosis in the study is plotted as “reported prior TB” in orange with corresponding color error margins for both trendlines. *C*, Three multivariable logistic regression models examining prior tuberculosis as the exposure upon frail and prefrail outcomes, relative to robust outcomes are reported as (i) unadjusted, (ii) age, sex, education and HIV status adjusted or (iii) fully adjusted models using odds ratios (OR) with 95% confidence intervals and *P* values estimated from Wald tests. The fully adjusted model includes age, sex, HIV status, education level, food insecurity, wealth index, country, diabetes, smoking, and alcohol use.).

Following adjustment for age, sex, educational attainment, and HIV status, prior tuberculosis was associated with twice the odds of frailty (OR 2.06, 95% CI 1.38–3.03) (Figure 1*C*). This association was robust to further adjustment for country, food insecurity, household wealth, diabetes, smoking and alcohol (OR 2.04, 95% CI 1.35–3.03). No clear relationship was observed between prefrailty and prior tuberculosis. No evidence of effect modification by age, sex, HIV status, or lung damage was detected (Supplementary Table 3).

## DISCUSSION

Our analysis identifies an important association between self-reported prior tuberculosis and frailty among older adults in Southern Africa. Tuberculosis survivors aged ≥40 years had approximately twice the odds of frailty compared with those with no tuberculosis history, after accounting for age, sex, HIV, diabetes, and socioeconomic differences. The strength of this relationship is almost certainly underestimated due to survivor bias.

Coinfection with HIV is a major driver of the tuberculosis epidemic in Southern Africa, and uncontrolled HIV can contribute to frailty [1]. As the highest HIV infection risk period for this age group also overlapped with the preantiretroviral era, many people falling ill with tuberculosis may have been living with untreated HIV, at a time when HIV-related mortality accounted for at least 60% of adult deaths [10]. Consequently, fewer people with comorbid prior tuberculosis have survived to older age, leaving proportionately more “healthy survivors” (relatively speaking) to be enrolled in our study. As such, reported prevalence of prior tuberculosis is lower than expected based upon estimates from annual case reports. Greater awareness of survivor bias and its potential to obscure the true impact of post-tuberculosis sequelae is an essential consideration for future studies.

The observed survival bias is only partially explained by HIV-associated mortality in our study. Even among HIV-negative tuberculosis survivors, the proportion of people with prior tuberculosis fails to accumulate across age groups as expected suggesting tuberculosis-associated adverse survival. Socioeconomic deprivation may possibly shape the relationship between tuberculosis and frailty. However, the persistent association between prior tuberculosis and frailty across age strata despite the observed survivor bias implies that our results are likely a conservative assessment of morbidity. Our analysis adjusted for food insecurity, education and household wealth, but residual confounding may in part explain the association between tuberculosis and frailty, given the established link between deprivation and frailty [7, 9]. We also cannot exclude reverse causation if frail/prefrail individuals are more susceptible to tuberculosis in the first instance. Other limitations include biased recall of prior tuberculosis, the cross-sectional design, which prevents causal inference, and self-reported exhaustion, weight loss and physical activity contributing to frailty definitions, although this approach has been validated in many settings [14]. Our prevalence estimates relied upon historic data only available for those aged ≥15 years and do not subtract losses to all-cause mortality. Findings specifically reflect settings with significant HIV epidemics and may not be as generalizable to regions with more mature aged care services.

Prospective longitudinal cohort studies, examining health outcomes among tuberculosis survivors beyond 2-years post-treatment are rare, severely limiting our understanding of vulnerability in later life. In one African setting, a 10-year retrospective follow-up of tuberculosis survivors revealed increased long-term all-cause mortality, but studies in high tuberculosis settings, which examine morbidity outcomes or causes of mortality are few [15].

The mechanisms linking prior tuberculosis to frailty are speculative, but established associations between frailty and other chronic respiratory diseases suggest chronic inflammation may be a mediator [6]. Parenchymal remodeling resulting from chronic inflammation could fuel increased metabolism, sarcopenia (loss of muscle mass and strength) and loss of appetite in people with tuberculosis [6]. As a result, tuberculosis-related cachexia and associated muscle loss, together with incomplete recovery and reduced physiological reserve, are biologically plausible mechanisms that could explain the exhaustion, low physical activity and unintended weight loss characterizing the frailty syndrome [2, 6]. As prior tuberculosis was consistently not associated with prefrailly across unadjusted and adjusted models, the observed association between tuberculosis history and frailty appears to be specific to the severe full frailty category, with no evidence of a graduated relationship. The full frailty syndrome maps closely with known post-tuberculosis sequelae described above, whereas prefrailty as a transitional state may reflect many pathways. The specificity of our finding to the full frailty syndrome arguably strengthens the biological plausibility of tuberculosis as a severe and lasting assault upon physiological resilience, not only productive of the subtle changes consistent with prefrailty and many other etiologies. Prospective longitudinal studies in high-incidence tuberculosis settings which routinely assess frailty, sarcopenia and physical performance among people with tuberculosis are needed to explore mechanisms and should include follow-up beyond the 2-year post-treatment window.

To our knowledge, increased frailty among adults with prior tuberculosis is not widely recognized, especially in Southern Africa. Given improved lifespans for coinfected tuberculosis survivors due to antiretroviral therapy, the region's high tuberculosis burden and the existence of interventions to treat and reverse frailty, these findings are relevant for public health and clinical care. Further research is needed to examine causality, investigate mechanisms and develop intervention strategies. The impact of tuberculosis on ageing has been underexplored in low- and middle-income settings and this association may be underestimated if survival bias is not considered. As longevity increases in Southern Africa, the long-term health of tuberculosis survivors needs to be specifically considered, to enable “healthy ageing” among an important, often socioeconomically disadvantaged but economically active, group of adults.

## Supplementary Material

jiag299_Supplementary_Data

## Supplementary Data

Supplementary materials are available at *The Journal of Infectious Diseases* online (http://jid.oxfordjournals.org/). Supplementary materials consist of data provided by the author that are published to benefit the reader. The posted materials are not copyedited. The contents of all supplementary data are the sole responsibility of the authors. Questions or messages regarding errors should be addressed to the author.
