# Supplementary material for: People With Prior Tuberculosis Have Accelerated Frailty and Differential Survival into Older Age: A Cross-sectional Analysis in Southern Africa
Source: J Infect Dis. 2026 Jun 12;234(4):e891–5. doi: 10.1093/infdis/jiag299 (PMC13668369; doi:10.1093/infdis/jiag299)
Supplement: Supplementary file 1 [file jiag299_supplementary_data.docx]

**SUPPLEMENT**

***People with prior tuberculosis have accelerated frailty and differential survival into older age: a cross-sectional analysis in Southern Africa***

**Supplementary table 1: Criteria and operational definition of frailty**

| **Criteria** | **Operationalisation** | |
| --- | --- | --- |
|  | **Urban Zimbabwe** | **Urban and rural South Africa** |
| **Weight loss** | Self-report to *“Have you, or those close to you, noticed that you have lost weight or become thinner in the last 12 months?”* | |
| **Exhaustion** | Self-reported based on one question of the Shona Symptom Questionnaire[1], which translates to mean *“I felt run down”.* | |
| **Low physical activity** | Self-reported based on the International Physical Activity Questionnaire - Short Form[2]. Metabolic equivalent (METS) minutes per week calculated[3] and converted to Kcal per week. Cutoffs propose by Fried et al[4] used: Men using <383 Kcals/week and Women using <270 Kcals/week | |
| **Low gait speed** (Mean measured time of two 4-metre (13.1 feet) walks) | Slowest 20% by sex and median height:  ***Men***  *Height ≤ 171.1cm: ≥ 6.3 seconds*  *Height > 171.1cm: ≥ 5.6 seconds*  ***Women***  *Height ≤ 159.7cm: ≥ 8.3 seconds*  *Height > 159.7cm: ≥ 6.6 seconds*  *†Plus, those with mobility challenges who did not complete the walk test, see footnote* | Slowest 20% by sex and median height:  ***Men***  *Height ≤ 167.9cm: ≥ 6.0 seconds*  *Height > 167.9cm: ≥ 6.0 seconds*  ***Women***  *Height ≤ 155.6cm: ≥ 7.4 seconds*  *Height > 155.6cm: ≥ 7.0 seconds*  *†Plus, those with mobility challenges who did not complete the walk test, see footnote* |
| **Low grip strength** (Maximal grip strength of six measures was used) | Cut-offs generated as part of the operationalisation of a sarcopenia definition in Africa using pooled data from The Gambia, Zimbabwe, and South Africa. Cut-offs based on 2 Standard Deviations below the mean grip in men and women: 20kg in women, 28kg in men | |
| †Further, those who did not complete the walk test and (i) self-reported immobility or mobility only with aids AND (ii) moderate to extreme problems with mobility AND (iii) 0 walking MET-minutes per week, were considered to meet the frailty criteria.  *Those who declined the grip strength test because they felt too weak, unsafe, or unable, had shaky hands or a stroke limiting use, were classified as having low grip strength. | | |

**Supplementary Methods**

***Estimation of expected cumulative prior tuberculosis prevalence***

The parent study protocol provides greater study design information.^11^ Using WHO case notification data based upon the total new and relapse cases (1980–2023, WHO), annual tuberculosis notification rates were calculated for adults aged ≥15 years in South Africa and Zimbabwe separately. These values were then divided by the corresponding ≥15 years population estimates available from Our World in Data for each calendar year.

For each age represented in the study sample, the calendar year in which each person would have turned 15 was identified. Cumulative lifetime tuberculosis risk was then estimated by summing the annual notification rates (expressed as percentages) from that year through to 2023, when the study took place. Our approach assumed that tuberculosis risk accumulates from age 15, reflecting both a greater tuberculosis epidemiological burden and exposure in those aged ≥15 years and the limitations of age-disaggregated available notification data at younger ages, which effectively prevented our ability to estimate cumulative risk before this age. For participants who turned 15 years before 1980 when data was reported, we extrapolated pre-1980 tuberculosis risk by applying a tuberculosis mean notification rate from the years 1980–1985 for each year of adult life prior to 1980.

Expected prevalence estimates were then standardised to the age composition of study participants within each 10-year age band. For each band, the proportion of study participants at each single year of age was used as a sampling weight, and the weighted sum of age-specific cumulative risks produced the expected prior tuberculosis prevalence for that age band.

Our methodological base assumptions reflected a preference for internal data consistency over time but created some limitations in our estimates. We excluded child tuberculosis by including risk for the ≥15 population only, likely resulting in a conservative underestimate of cumulative tuberculosis survivors, particularly for older age groups. We also used notification data counts that combined all new and relapse cases. In settings with high recurrence rates such as South Africa and Zimbabwe, the same individual may contribute to WHO case counts in multiple years, inflating the estimated number of unique tuberculosis survivors. Finally, expected prevalence estimates were not adjusted for all-cause mortality. Since tuberculosis elevates all-cause mortality for several years post-infection,^6^ disentangling tuberculosis-attributable deaths from unrelated mortality was not considered feasable with available data (or knowledge within the field).

**Supplementary table 2. Frailty and cognition factors associated with prior tuberculosis in older adults stratified by sex**

|  | **Total** | | | | **Male** | | | | **Female** | | | |
| --- | --- | --- | --- | --- | --- | --- | --- | --- | --- | --- | --- | --- |
| **Characteristic** | **Overall***^1^* | **No prior TB***^1^* | **Prior TB***^1^* | **p-value***^2^* | **Overall***^1^* | **No prior TB***^1^* | **Prior TB***^1^* | **p-value***^2^* | **Overall***^1^* | **No prior TB***^1^* | **Prior TB***^1^* | **p-value***^2^* |
| **Sociodemographic characteristics** |  |  |  |  |  |  |  |  |  |  |  |  |
| Age group (years) |  |  |  | <0.001 |  |  |  | <0.001 |  |  |  | <0.001 |
| 40–54 | 1,039/2,995 (34.7%) | 907/2,724 (33.3%) | 132/271 (48.7%) |  | 521/1,405 (37.1%) | 442/1,236 (35.8%) | 79/169 (46.7%) |  | 518/1,590 (32.6%) | 465/1,488 (31.3%) | 53/102 (52.0%) |  |
| 55–64 | 768/2,995 (25.6%) | 672/2,724 (24.7%) | 96/271 (35.4%) |  | 397/1,405 (28.3%) | 333/1,236 (26.9%) | 64/169 (37.9%) |  | 371/1,590 (23.3%) | 339/1,488 (22.8%) | 32/102 (31.4%) |  |
| ≥65 | 1,188/2,995 (39.7%) | 1,145/2,724 (42.0%) | 43/271 (15.9%) |  | 487/1,405 (34.7%) | 461/1,236 (37.3%) | 26/169 (15.4%) |  | 701/1,590 (44.1%) | 684/1,488 (46.0%) | 17/102 (16.7%) |  |
| Country |  |  |  | <0.001 |  |  |  | <0.001 |  |  |  | <0.001 |
| South Africa | 1,890/2,995 (63.1%) | 1,676/2,724 (61.5%) | 214/271 (79.0%) |  | 869/1,405 (61.9%) | 736/1,236 (59.5%) | 133/169 (78.7%) |  | 1,021/1,590 (64.2%) | 940/1,488 (63.2%) | 81/102 (79.4%) |  |
| Zimbabwe | 1,105/2,995 (36.9%) | 1,048/2,724 (38.5%) | 57/271 (21.0%) |  | 536/1,405 (38.1%) | 500/1,236 (40.5%) | 36/169 (21.3%) |  | 569/1,590 (35.8%) | 548/1,488 (36.8%) | 21/102 (20.6%) |  |
| Education |  |  |  | <0.001 |  |  |  | 0.019 |  |  |  | <0.001 |
| Primary | 1,120/2,995 (37.4%) | 1,061/2,724 (39.0%) | 59/271 (21.8%) |  | 438/1,405 (31.2%) | 401/1,236 (32.4%) | 37/169 (21.9%) |  | 682/1,590 (42.9%) | 660/1,488 (44.4%) | 22/102 (21.6%) |  |
| Secondary | 1,862/2,995 (62.2%) | 1,650/2,724 (60.6%) | 212/271 (78.2%) |  | 965/1,405 (68.7%) | 833/1,236 (67.4%) | 132/169 (78.1%) |  | 897/1,590 (56.4%) | 817/1,488 (54.9%) | 80/102 (78.4%) |  |
| Unknown | 13/2,995 (0.4%) | 13/2,724 (0.5%) | 0/271 (0.0%) |  | 2/1,405 (0.1%) | 2/1,236 (0.2%) | 0/169 (0.0%) |  | 11/1,590 (0.7%) | 11/1,488 (0.7%) | 0/102 (0.0%) |  |
| Wealth Index |  |  |  | 0.007 |  |  |  | 0.003 |  |  |  | 0.5 |
| Low | 1,151/2,995 (38.4%) | 1,049/2,724 (38.5%) | 102/271 (37.6%) |  | 540/1,405 (38.4%) | 473/1,236 (38.3%) | 67/169 (39.6%) |  | 611/1,590 (38.4%) | 576/1,488 (38.7%) | 35/102 (34.3%) |  |
| Middle | 1,208/2,995 (40.3%) | 1,079/2,724 (39.6%) | 129/271 (47.6%) |  | 565/1,405 (40.2%) | 483/1,236 (39.1%) | 82/169 (48.5%) |  | 643/1,590 (40.4%) | 596/1,488 (40.1%) | 47/102 (46.1%) |  |
| High | 636/2,995 (21.2%) | 596/2,724 (21.9%) | 40/271 (14.8%) |  | 300/1,405 (21.4%) | 280/1,236 (22.7%) | 20/169 (11.8%) |  | 336/1,590 (21.1%) | 316/1,488 (21.2%) | 20/102 (19.6%) |  |
| Food Insecure | 1,555/2,995 (51.9%) | 1,378/2,724 (50.6%) | 177/271 (65.3%) | <0.001 | 721/1,405 (51.3%) | 609/1,236 (49.3%) | 112/169 (66.3%) | <0.001 | 834/1,590 (52.5%) | 769/1,488 (51.7%) | 65/102 (63.7%) | 0.018 |
| BMI category |  |  |  | <0.001 |  |  |  | <0.001 |  |  |  | 0.023 |
| Underweight <18.5Kg/m2 | 147/2,958 (5.0%) | 116/2,689 (4.3%) | 31/269 (11.5%) |  | 125/1,394 (9.0%) | 96/1,226 (7.8%) | 29/168 (17.3%) |  | 22/1,564 (1.4%) | 20/1,463 (1.4%) | 2/101 (2.0%) |  |
| Healthy weight 18.5-<25.0Kg/m2 | 1,016/2,958 (34.3%) | 893/2,689 (33.2%) | 123/269 (45.7%) |  | 746/1,394 (53.5%) | 644/1,226 (52.5%) | 102/168 (60.7%) |  | 270/1,564 (17.3%) | 249/1,463 (17.0%) | 21/101 (20.8%) |  |
| Overweight 25-<30Kg/m2 | 710/2,958 (24.0%) | 649/2,689 (24.1%) | 61/269 (22.7%) |  | 333/1,394 (23.9%) | 306/1,226 (25.0%) | 27/168 (16.1%) |  | 377/1,564 (24.1%) | 343/1,463 (23.4%) | 34/101 (33.7%) |  |
| Obese ≥30Kg/m2 | 1,085/2,958 (36.7%) | 1,031/2,689 (38.3%) | 54/269 (20.1%) |  | 190/1,394 (13.6%) | 180/1,226 (14.7%) | 10/168 (6.0%) |  | 895/1,564 (57.2%) | 851/1,463 (58.2%) | 44/101 (43.6%) |  |
| **Comorbid disease** |  |  |  |  |  |  |  |  |  |  |  |  |
| Living with HIV | 928/2,995 (31.0%) | 747/2,724 (27.4%) | 181/271 (66.8%) | <0.001 | 430/1,405 (30.6%) | 328/1,236 (26.5%) | 102/169 (60.4%) | <0.001 | 498/1,590 (31.3%) | 419/1,488 (28.2%) | 79/102 (77.5%) | <0.001 |
| Diabetes (type 2) | 434/2,995 (14.5%) | 412/2,724 (15.1%) | 22/271 (8.1%) | 0.002 | 157/1,405 (11.2%) | 143/1,236 (11.6%) | 14/169 (8.3%) | 0.2 | 277/1,590 (17.4%) | 269/1,488 (18.1%) | 8/102 (7.8%) | 0.008 |
| Prior or current cancer | 27/2,889 (0.9%) | 19/2,640 (0.7%) | 8/249 (3.2%) | 0.001 | 14/1,357 (1.0%) | 10/1,204 (0.8%) | 4/153 (2.6%) | 0.063 | 13/1,532 (0.8%) | 9/1,436 (0.6%) | 4/96 (4.2%) | 0.007 |
| Lung damage | 27/2,905 (0.9%) | 19/2,653 (0.7%) | 8/252 (3.2%) | 0.001 | 14/1,367 (1.0%) | 10/1,212 (0.8%) | 4/155 (2.6%) | 0.064 | 13/1,538 (0.8%) | 9/1,441 (0.6%) | 4/97 (4.1%) | 0.007 |
| Neurodegenerative condition | 168/2,995 (5.6%) | 147/2,724 (5.4%) | 21/271 (7.7%) | 0.11 | 90/1,405 (6.4%) | 78/1,236 (6.3%) | 12/169 (7.1%) | 0.7 | 78/1,590 (4.9%) | 69/1,488 (4.6%) | 9/102 (8.8%) | 0.058 |
| **'Lifestyle' factors** |  |  |  |  |  |  |  |  |  |  |  |  |
| Current or former smoking | 717/2,993 (24.0%) | 603/2,723 (22.1%) | 114/270 (42.2%) | <0.001 | 609/1,403 (43.4%) | 512/1,235 (41.5%) | 97/168 (57.7%) | <0.001 | 108/1,590 (6.8%) | 91/1,488 (6.1%) | 17/102 (16.7%) | <0.001 |
| Alcohol consumption | 820/2,986 (27.5%) | 691/2,715 (25.5%) | 129/271 (47.6%) | <0.001 | 643/1,403 (45.8%) | 543/1,234 (44.0%) | 100/169 (59.2%) | <0.001 | 177/1,583 (11.2%) | 148/1,481 (10.0%) | 29/102 (28.4%) | <0.001 |
| **Frailty factors** |  |  |  |  |  |  |  |  |  |  |  |  |
| Frailty category |  |  |  | 0.4 |  |  |  | 0.4 |  |  |  | 0.3 |
| Frail | 409/2,995 (13.7%) | 369/2,724 (13.5%) | 40/271 (14.8%) |  | 158/1,405 (11.2%) | 135/1,236 (10.9%) | 23/169 (13.6%) |  | 251/1,590 (15.8%) | 234/1,488 (15.7%) | 17/102 (16.7%) |  |
| Pre-frail | 1,766/2,995 (59.0%) | 1,600/2,724 (58.7%) | 166/271 (61.3%) |  | 805/1,405 (57.3%) | 706/1,236 (57.1%) | 99/169 (58.6%) |  | 961/1,590 (60.4%) | 894/1,488 (60.1%) | 67/102 (65.7%) |  |
| Robust | 820/2,995 (27.4%) | 755/2,724 (27.7%) | 65/271 (24.0%) |  | 442/1,405 (31.5%) | 395/1,236 (32.0%) | 47/169 (27.8%) |  | 378/1,590 (23.8%) | 360/1,488 (24.2%) | 18/102 (17.6%) |  |
| *^1^*n/N (%) | | | | | | | | | | | | |
| *^2^*Pearson's Chi-squared test or Fisher’s exact test | | | | | | | | | | | | |

**Supplementary table 3: Stratified analysis of the association between prior tuberculosis and frailty, by sex, age group, HIV status and lung damage using univariate and multivariable logistic regression**

| **Subgroup** | **N** | **Unadjusted OR** | **Age, Sex, Education & HIV Adjusted OR** | **Fully Adjusted OR** | **Interaction**  ***p*-value** |
| --- | --- | --- | --- | --- | --- |
| **Sex** | |  |  | | |
| *Male* | 1416 | 1.22 (0.86–1.75) | 1.52 (1.06–2.23) | 1.53 (1.05–2.25) | 0.351 |
| *Female* | 1596 | 1.49 (0.91–2.59) | 2.07 (1.23–3.65) | 2.03 (1.19–3.62) |  |
| **Age group** | |  |  | | |
| *40–54* | 1045 | 1.56 (1.05–2.38) | 1.75 (1.15–2.70) | 1.70 (1.11–2.65) | 0.752 |
| *55–64* | 772 | 1.39 (0.88–2.28) | 1.41 (0.87–2.35) | 1.34 (0.81–2.27) |  |
| *≥65* | 1195 | 2.04 (0.81–6.87) | 2.59 (1.01–8.79) | 2.44 (0.94–8.33) |  |
| **Living with HIV** | |  |  | | |
| *No* | 2079 | 1.28 (0.78–2.21) | 1.76 (1.05–3.06) | 1.71 (1.02–3.00) | 0.351 |
| *Yes* | 933 | 1.47 (1.02–2.14) | 1.65 (1.14–2.42) | 1.59 (1.09–2.36) |  |
| **Lung damage** | |  |  | | |
| *No* | 2890 | 1.22 (0.90–1.67) | 1.67 (1.22–2.32) | 1.68 (1.22–2.34) | 0.959 |
| *Yes* | 117 | 1.36 (0.49–4.44) | 3.14 (1.00–11.45) | 1.80 (0.42–8.54) |  |
| *^1^*Partially adjusted model includes age, sex, HIV status, and education (excluding stratifying variable). Fully adjusted model additionally includes food insecurity, country, diabetes, smoking, alcohol use, and wealth index (excluding stratifying variable). Lung damage excluded from all adjustment sets as a potential mediator; variable includes known asthma and chronic obstructive pulmonary disease. Interaction p-value from likelihood ratio test comparing partially adjusted models with and without interaction term. OR = Odds Ratio; CI = Confidence Interval. | | | | | |

**Supplementary references**

1. Patel V, Simunyu E, Gwanzura F, Lewis G, Mann A. The Shona Symptom Questionnaire: the development of an indigenous measure of common mental disorders in Harare. Acta Psychiatr Scand. 1997;95(6):469-75. doi: 10.1111/j.1600-0447.1997.tb10134.x. PubMed PMID: 9242841.

2. Craig C, Marshall A, Sjostrom M, Bauman A, Lee P, Macfarlane D, et al. International physical activity questionnaire-short form. J Am Coll Health. 2017;65(7):492-501.

3. Forde C. Scoring the international physical activity questionnaire (IPAQ). University of Dublin. 2018;3.

4. Fried LP, Tangen CM, Walston J, Newman AB, Hirsch C, Gottdiener J, et al. Frailty in older adults: evidence for a phenotype. J Gerontol A Biol Sci Med Sci. 2001;56(3):M146-56. doi: 10.1093/gerona/56.3.m146. PubMed PMID: 11253156.

5. Theou O, Cann L, Blodgett J, Wallace LMK, Brothers TD, Rockwood K. Modifications to the frailty phenotype criteria: Systematic review of the current literature and investigation of 262 frailty phenotypes in the Survey of Health, Ageing, and Retirement in Europe. *Ageing Res Rev* 2015; **21**: 78–94.

6. Romanowski K, Baumann B, Basham CA, Ahmad Khan F, Fox GJ, Johnston JC. Long-term all-cause mortality in people treated for tuberculosis: a systematic review and meta-analysis. *The Lancet Infectious Diseases* 2019; **19**: 1129–37.
